# Acute human brain responses to intracortical microelectrode arrays: challenges and future prospects

**DOI:** 10.3389/fneng.2014.00024

**Published:** 2014-07-21

**Authors:** Eduardo Fernández, Bradley Greger, Paul A. House, Ignacio Aranda, Carlos Botella, Julio Albisua, Cristina Soto-Sánchez, Arantxa Alfaro, Richard A. Normann

**Affiliations:** ^1^Bioengineering Institute, Miguel Hernández University of ElcheElche, Spain; ^2^CIBER-BBNZaragoza, Spain; ^3^School of Biological and Health Systems Engineering, Arizona State UniversityTempe, AZ, USA; ^4^Department of Neurosurgery, University of UtahSalt Lake City, UT, USA; ^5^Department of Pathology, Hospital General UniversitarioAlicante, Spain; ^6^Department of Neurosurgery, Hospital La FeValencia, Spain; ^7^Department of Neurosurgery, Fundación Jimenez Díaz and Hospital Rey Juan CarlosMadrid, Spain; ^8^Department of Bioengineering, University of UtahSalt Lake City, UT, USA

**Keywords:** neural prosthesis, intracortical microelectrode, *in vivo* recording, biocompatibility, neurosurgery

## Abstract

The emerging field of neuroprosthetics is focused on the development of new therapeutic interventions that will be able to restore some lost neural function by selective electrical stimulation or by harnessing activity recorded from populations of neurons. As more and more patients benefit from these approaches, the interest in neural interfaces has grown significantly and a new generation of penetrating microelectrode arrays are providing unprecedented access to the neurons of the central nervous system (CNS). These microelectrodes have active tip dimensions that are similar in size to neurons and because they penetrate the nervous system, they provide selective access to these cells (within a few microns). However, the very long-term viability of chronically implanted microelectrodes and the capability of recording the same spiking activity over long time periods still remain to be established and confirmed in human studies. Here we review the main responses to acute implantation of microelectrode arrays, and emphasize that it will become essential to control the neural tissue damage induced by these intracortical microelectrodes in order to achieve the high clinical potentials accompanying this technology.

## Introduction

Since 1990, the field of neuroprosthetics has grown at an ever increasing rate. Although current research is also pursuing non-invasive techniques to acquire signals from the human brain, important scientific and clinical information has been gained from *in vivo* experiments with microelectrode arrays able to record or stimulate the nervous system with the aim of improving or replacing motor or sensory abilities that have been lost due to disease or injury. For example, auditory brainstem implants are being used to restore auditory function (Merkus et al., 2014); deep brain stimulators have been implanted successfully in patients for control of motor disorders, tremor, dystonia and chronic pain (Beitz, 2014; Nardone et al., 2014) and micro-array type devices have been implanted in artificial vision systems (Grill et al., 2009; Hatsopoulos and Donoghue, 2009; Normann et al., 2009; Fernandez and Hofmann, 2011). Moreover advances in prosthetic limbs and brain-machine interfaces are now providing hope of increased mobility and independence for amputees and paralyzed patients (Raspopovic et al., 2014) and intracortical micro-electrodes are being used to study epileptiform discharges in cases of intractable epilepsy (Weiss et al., 2013a,b). As more and more patients have benefited from this approach, the interest in neural interfaces has grown significantly and a new generation of penetrating microelectrode arrays are providing unprecedented access to the neurons of the central nervous system (CNS).

These studies have shown the utility of microelectrode recordings to resolve neural activity at the single neuron level and to interpret brain derived commands for fine-grained control of movements and translation of these signals into command signals that are able to control external devices (Hochberg et al., 2006; Andersen et al., 2010; Rao and Donoghue, 2014). However most penetrating microelectrode arrays currently have maximum *in vivo* lifetimes from several months to a few years (Suner et al., 2005; Prasad et al., 2012; Barrese et al., 2013). Therefore much work still needs to be done before these penetrating microelectrodes can be used for many clinical purposes. In this work we focused on the acute effects of implantation of penetrating microelectrode arrays in the human brain, emphasizing the relevance of surgical techniques and biocompatibility approaches to control the neural tissue damage induced by these intracortical probes in order to achieve the therapeutic benefits envisioned by these neural interfaces.

## Current multielectrode-array technologies

Technology partially achieved during the development of cardiac pacemakers has been successfully used in many other applications of implanted neural prostheses. During these years great efforts have been made to develop penetrating multi-electrode arrays with dimensions similar to the cortical neurons they target for recording or stimulation and that are able to maintain a stable signal in the CNS. The two main dominant approaches are multiple insulated metal microwires (Nicolelis and Lebedev, 2009; Freire et al., 2011; Carmena, 2013) and penetrating microelectrode arrays fabricated with micro-electro-mechanical-system technologies. These devices use various substrate materials that can either be flexible and based on polymers (Rousche et al., 2001; Chen et al., 2009; Kozai and Kipke, 2009; Hassler et al., 2011) or rigid such as the Utah Electrode Array (UEA; Normann, 2007), the Michigan array (Seymour and Kipke, 2007) or the NeuroProbes arrays (Neves, 2007; Calixto et al., 2013). However today, only a few of these devices are commercially available and do not yet exist as commercial, wireless, implantable, many-channel devices that can provide reliable recording and stimulation for many years.

An ideal neural interface would consist of an array containing many microelectrodes, which can “listen” and “talk” to still-functioning parts of the brain enabling bi-directional communication with ensembles of neurons. Each microelectrode would either record the electrical activity of a small population of neurons surrounding it or, when electrical current is passed through the electrode, activate small population of neurons. This approach has been difficult to realize because of the acute and chronic inflammatory reactions that can induce significant changes at the brain-electrode interface. Thus all neural probes need a stable electronic/neural interface that enables selective recording and/or activation of specific groups of neurons without deterioration of the electrodes or surrounding neural tissue. Consequently three areas have to be considered: the “*biosafety*”, the “*biofunctionality*” and the “*biostability*” of the devices. Biosafety means that the microelectrodes do not harm the brain tissue in any significant way, biofunctionality is related to the ability of the microelectrodes to perform their intended function, and biostability means that the whole microelectrode array must not be susceptible to attack of biological fluids, proteases, macrophages or any metabolic byproducts (Marin and Fernandez, 2010). In addition successful devices should manifest “biotolerability” or the ability of the multielectrode-array to reside in the CNS for long periods of time. All these considerations impose extreme demands on stability and function of neural implants and place unique constraints on the architecture, materials, and surgical techniques used in the application of intracortical microelectrodes.

## Surgical procedures for implantation of intracortical microelectrodes arrays

**Figure 1:**
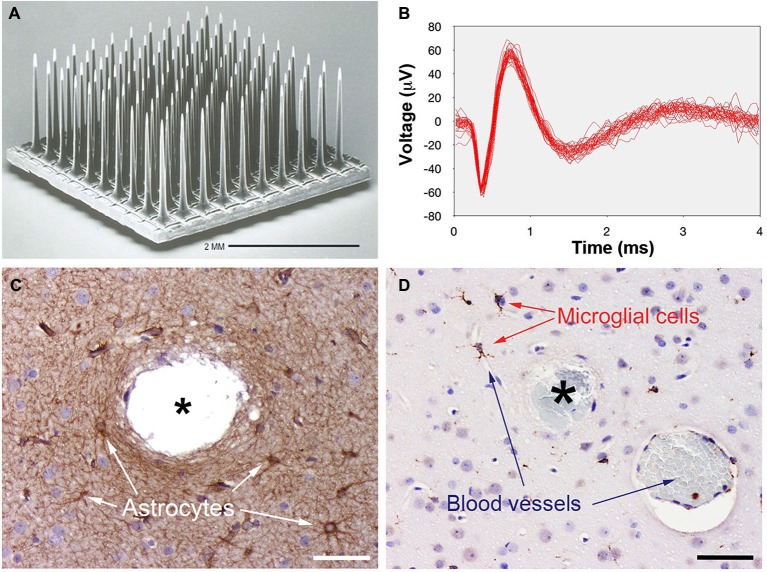
**Photographs showing the silicon-based Utah Electrode Array (UEA) and representative results of its implantation into human cortex.** **(A)** Scanning electron micrograph of the UEA. **(B)** Single-unit responses recorded with the UEA from human temporal cortex (47 superimposed traces). **(C)** Astrocytes, labeled here with anti-glial fibrillary acidic protein antibody (GFAP) increase the thickness of their main processes, especially around electrode tracks (asterisk). **(D)** Resident microglial cells and blood-borne macrophages, labeled here with anti-CD45 antibody, become activated and migrate toward the electrodes. Note the electrode track filled with blood cells (asterisk) and a nearby blood vessel. Calibration bars = 50 μm.

Microelectrode arrays aimed to simultaneously record or stimulate neuronal populations have been successfully used in many animal models including non-human primates (Normann, 2007). However the only intracortical microelectrode array that has been FDA approved for long-term human studies is the UEA (Nordhausen et al., 1996). This microelectrode array consists of 100 small diameter silicon microneedles, built on a square grid with 400 μm spacing, that were designed to be inserted into the cerebral cortex to a depth of 1.5 mm, the level of thalamic input to the cerebral cortex (Figure 1A).

The UEA has been used in BrainGate clinical trials (Hochberg et al., 2006; Homer et al., 2013; Perge et al., 2013) and in research with epilepsy patients (Normann et al., 2009; Truccolo et al., 2011; Weiss et al., 2013a,b), and we have performed a number of preliminary experiments designed to establish the safety of the implantation procedures (House et al., 2006). These experiments were performed in persons suffering from epilepsy or brain tumors that had to undergo a surgical resection of a brain region. Briefly after exposure of the implantation site using standard neurosurgical equipment and procedures, the pia arachnoid was cut to allow access to the surface of the cerebral cortex. Even though the individual electrodes of the UEA are very sharp (with radius curvatures typically on the order of a few microns) we found that trying to push 100 electrodes into cortical tissues only depressed the surface of the tissue and resulted in only partial insertion of the electrode array. A pneumatically actuated precision instrument that allows the complete and safe insertion of the array in under 200 ms was developed to circumvent this difficulty (Rousche and Normann, 1992). Interestingly we found that it was necessary to fine adjust the parameters of the pneumatic inserter depending of the age of the patients, and that it was easier to get a complete insertion in young than in aged patients using low insertion pressures (around 15–20 psi).

## Human acute reactive responses around implanted microelectrodes

**Figure 2:**
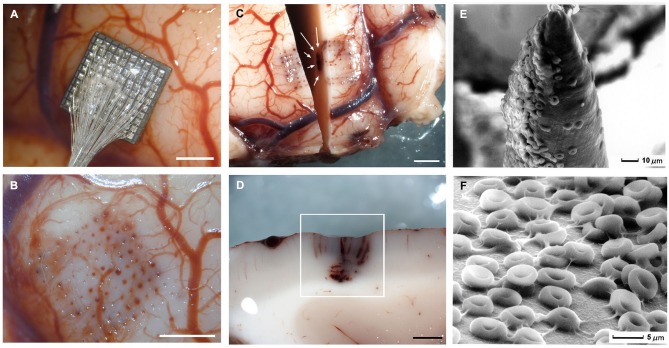
**Gross specimens of human temporal lobe implantations and scanning micrographs of the surface of the Utah Electrode Array after acute implantation in human brain**. **(A)** Placement of an electrode array in temporal cortex. **(B)** Once the array has been removed there are some evident microhemorrhages. **(C)** Horizontal section showing blood in the outermost electrode tracks and petechial hemorrhages (white arrows) located below the tip of the electrodes. **(D)** Detail of the petechial hemorrhages. **(E)** Scanning electron micrograph of an electrode tip. Many red blood cells appear in close contact with electrode materials. **(F)** Detail of the red blood cells on the surface of the microelectrodes. Calibration bars A, B, C and D= 2 mm.

Implantation of any neural probe is always a traumatic procedure that implies some local damage of neurons, vasculature and other cells (Figures 1C,D). Thus, when a neural probe is inserted into the brain, some neurons and glial cells are killed or injured during insertion, blood vessels are disrupted, and the blood-brain barrier is compromised (Figures 2A,B). As a result, with most of our microelectrode array insertions in human cortex, we typically observed interstitial microhemorrhages emanating from the electrode tracks that extended in one or more directions. These microhemorrhages were limited to within a few millimeters of the microelectrode tracks although these were more evident around the edges of the arrays. This damage seemed to result from a combination of the numerous blood vessels encountered in the path of the penetrating electrodes plus some mechanical damage of small capillaries, especially at the borders of the array. Furthermore although the insertion of the UEA did not result in clinically relevant hemorrhages, some times we found petechial hemorrhages located below the tips of the electrodes (Figures 2C,D).

These microhemorrhages usually stopped spontaneously or after gentle irrigation with normal saline and we do not have any case where we had to remove the array due to the bleeding. However, due to this small bleeding various serum plasma proteins and blood cells stick on the surface of the electrodes and trigger complement system activation, platelet activation, clot formation and a large network of changes including the release of cytokines, invasion of blood-borne macrophages and edema. Figure 2E shows an example of the tip of an electrode after explantation. Many red blood cells are seen in close contact with electrode materials (see Figure 2F). Thus, microhemorrhaging is an important issue that should be taken into account to reduce the adverse nature of the reactions and maintain an ideal environment for the microelectrodes.

Array implantation also causes an early activation and migration of microglial cells towards the microelectrodes, which could reflect highly specific interactions mediated by selectins, integrins, cytokines and carbohydrate-binding receptors (Kaur et al., 2010; Linnartz et al., 2012). These microglial cells are very sensitive to pathological conditions, even if they respond only to variations in local extracellular ionic concentrations (Kreutzberg, 1996; Freire et al., 2011) and we have found that there is a rapid activation, in a matter of minutes, of these cells in human brain. Figure 1D shows an example in an experiment in which the UEA was kept in place for <10 min.

Usually these scavenger cells form a network of immune alert resident macrophages with a capacity for immune surveillance and control. Therefore the initial activation and migration of microglial cells are likely beneficial and include production of neurotrophic substances and cell adhesion molecules, which support injured neurons and appear necessary for restorative events to take place (Eddleston and Mucke, 1993). However, this largely beneficial initial phase can result in a more adverse long-term response that is dependent on the extent of the injury. Consequently more effort is needed to control these responses and ensure the function of the implant without eliciting either structural, cellular or metabolic changes that compromise the microelectrode array performance and/or that result in tissue degeneration around the implanted microelectrodes.

## Electrophysiological recordings

As has been shown from experiments in rodents and non-human primates (Oliveira and Dimitrov, 2008), we found that the quality of the surgical implantation procedure plays a major role for tissue preservation and for the outcome of recordings. Our experimental results have demonstrated that high-quality microelectrode recordings from cerebral cortical neurons can be consistently obtained in both acute (intraoperative) and short term chronic (in an epilepsy monitoring unit) settings. An example of action potentials recorded with a UEA in the temporal cortex of a patient with medically intractable epilepsy is shown in Figure 1B. Single units are easily identifiable with a quality similar to that observed in rat, cat or non-human primate recordings. Furthermore, most of the presently available intracortical microelectrode arrays (including the UEA) allow recording of local field potentials that are very reliable and that can be used to detect changes in recording quality over time. Also, recording quality is affected by other surgical procedures: most drugs used for the induction and maintenance of general anesthesia decrease action potential firing rates and the information processing capacity in the neocortex (Hanrahan et al., 2013). In addition local fluctuation in K^+^ and Ca^+^ due to acute initial trauma can induce the silencing of neurons in the proximity of microelectrodes. Consequently recording sessions are usually initiated between 3–10 days after implantation.

The ability of intracortical microelectrodes arrays such as the UEA to record single units from human cortical neurons demonstrates that the implantation can be done without major complications, and emphasizes its potential role for studying encoding and processing of sensory and motor information by large neuronal ensembles. However significant variations in spike waveforms across time have been reported (Parker et al., 2011). These variations could reflect the initial acute response that can lead to chronic inflammatory reactions. Such reactions can negatively impact neurons and microelectrodes via induction of glial proliferation that produces a slow progressive decline in spike amplitude and in the number of viable channels. Furthermore recordings from individual neurons made with penetrating microelectrodes are often lost but then recover, possibly because of micro-motion of the device relative to the neural tissue (Parker et al., 2011). This problem can be mitigated with sophisticated action potential identification and daily recalibration.

## Challenges and future work

Intracortical microelectrode arrays have several advantages over traditional macroelectrode brain electrophysiological techniques such as their ability to selectively access individual or small groups of cortical neurons and the possibility to deliver relevant spatio-temporal patterns of stimulation. This highlights their potential to restore some lost neural function through selective electrical stimulation or by recording activity from selected populations of neurons. Furthermore the procedures for the implantation of penetrating microelectrodes are straightforward and well within the reach of most well-trained neurosurgeons. Altogether these results suggest that intracortical microelectrodes could form the basis of new neuroprosthetic devices for treating many disorders of the nervous system. For example they are presently being used to obtain volitional command signals from primary motor cortex of people with high cervical spinal injuries or to provide a limited but useful sense of vision in profoundly blind. However the presence of acute inflammatory reactions that can lead to chronic inflammatory reactions affecting both the neural tissue and the surface of the microelectrodes must be better understood and new approaches pursued to mitigate these reactions. Controlling and reducing the neural tissue damage induced by these intracortical microelectrodes should aid in keeping these devices biologically and electrically viable for many years.

Factors affecting brain tissue reactions to intracortical microelectrodes include the mechanical trauma during insertion, implantation method, biological acute responses and the physical properties of the microelectrodes. Because the brain is so richly vascularized, we have observed that there is inevitable bleeding associated with electrode array insertion and this suggests that bleeding is an important issue that should be taken into account to reduce the neural tissue damage induced by the microelectrodes. Moreover the reliability of recording the same spiking activity over long time periods still remains an unresolved problem that must be solved in longer term human studies before these penetrating microelectrodes can be used for some clinical purposes.

In this framework, important criteria for clinical success are tissue and microelectrode preservation and the maintenance of a stable signal in the CNS for the longest time possible. Remarkable progress has been reported in the use of these penetrating microelectrode arrays, but the electrode-tissue interface remains one of the major obstacles. Intracortical microelectrodes need a stable electronic/neural interface that facilitates selective recording and/or activation of specific groups of neurons without damage to the electrodes or surrounding neural tissues. Consequently it is essential to better understand the signals that lead to neuroglial activation in human brain and to create a targeted intervention strategy to prevent or at least to control this response.

Progress in this area relies on scientists being able to integrate and utilize methodologies from disparate disciplines, such as biomedical engineering, biomaterials, neuroscience, neurology, neurosurgery, information and communication technologies, molecular biology, etc. We are optimistic that with an emphasis on collaboration and a concerted push for additional clinical trials, these technologies will form the basis of devices and therapies that will substantially reduce the burden of lost neurological functions. However progress will be incremental and researchers must avoid creating false expectations that could damage the credibility of these new technologies.

## Conflict of interest statement

The authors declare that the research was conducted in the absence of any commercial or financial relationships that could be construed as a potential conflict of interest.
